# The Discovery of New Antilisterial Proteins From *Paenibacillus polymyxa* Kp10 via Genome Mining and Mass Spectrometry

**DOI:** 10.3389/fmicb.2020.00960

**Published:** 2020-07-02

**Authors:** Nur Fadhilah Khairil Mokhtar, Amalia Mohd Hashim, Irwan Hanish, Aisyah Zulkarnain, Raja Mohd Hafidz Raja Nhari, Asmahani Azira Abdul Sani, Sahar Abbasiliasi, Arbakariya Ariff, Shuhaimi Mustafa, Raha Abdul Rahim

**Affiliations:** ^1^Department of Cell and Molecular Biology, Faculty of Biotechnology and Biomolecular Sciences, Universiti Putra Malaysia, Selangor, Malaysia; ^2^Halal Products Research Institute, Putra Infoport, Universiti Putra Malaysia, Selangor, Malaysia; ^3^Department of Microbiology, Faculty of Biotechnology and Biomolecular Sciences, Universiti Putra Malaysia, Selangor, Malaysia; ^4^Mass Spectrometry Technology Section, Malaysia Genome Institute, National Institute of Biotechnology Malaysia, Kajang, Malaysia; ^5^Bioprocessing and Biomanufacturing Research Centre, Faculty of Biotechnology and Biomolecular Sciences, Universiti Putra Malaysia, Selangor, Malaysia; ^6^Chancellory, Universiti Teknikal Malaysia Melaka, Malacca, Malaysia

**Keywords:** antilisterial protein, genome mining, heterologous protein expression, mass spectrometry, *Paenibacillus polymyxa* Kp10, *Listeria monocytogenes*

## Abstract

The inhibitory properties of novel antimicrobial proteins against food-borne pathogens such as *Listeria monocytogenes* offer extensive benefits to the food and medical industries. In this study, we have identified antimicrobial proteins from a milk curd–derived bacterial isolate that exhibits antilisterial activity using genome mining and mass spectrometry analysis. The analysis of the draft genome sequence identified the isolate as *Paenibacillus polymyxa* Kp10, and predicted the presence of antimicrobial paenibacillin, paenilan, paeninodin, sactipeptides, thiazole-oxazole modified microcin, and histone-like DNA binding protein HU encoded in its genome. Interestingly, nanoLC-MS/MS analysis identified two histone-like DNA binding proteins HU as predicted *in silico* earlier, exhibiting antilisterial activity. Additionally, translation initiation factor IF-1 and 50S ribosomal protein L29 were also discovered by the mass spectrometry in the active fractions. The antilisterial activity of the four proteins was verified through heterologous protein expression and antimicrobial activity assay *in vitro*. This study has identified structural regulatory proteins from *Paenibacillus* possessing antilisterial activity with potential future application in the food and medical industries.

## Introduction

Food spoilage caused by pathogenic microbes such as *Listeria monocytogenes* (*L. monocytogenes*) is a common problem, imposing serious detrimental economic effects. The pathogen *L. monocytogenes* has become part of most foodborne outbreaks due to its ubiquitous nature and ability to survive in extreme conditions (Angelo et al., 2017). Its ability to form a biofilm on the surface of food processing equipment and its development of resistance to the standard sterilizing procedure have imposed further burdens on the food industry (Skowron et al., 2018). Common food preservatives to eradicate pathogen contamination include acetic acid, potassium acetate, benzoic acid, benzoates, and nitrites and nitrates. However, chemically derived preservatives have been shown to compromise human health in long-term use. For instance, the cytotoxic and mutagenic activity of sodium benzoate, a common preservative in dairy products, has been found in lymphocytes (Pongsavee, 2015). In addition, the reaction of nitrites in the form of sodium and potassium nitrite with secondary amines has been postulated to contribute to the formation of carcinogenic nitrosamines molecule (Carocho et al., 2014). Antimicrobial proteins are considered greener and safer, as they are biologically produced (Bagenda and Yamazaki, 2007). Therefore, the use of antimicrobial proteins in food and medical industries has increased in the past decades due to their inhibitory activity against food-borne pathogens and multidrug-resistant bacteria.

Bacteriocin, a ribosomally synthesized antimicrobial peptide, is a common antimicrobial protein used in the food and medical fields (Bagenda and Yamazaki, 2007; Yang et al., 2014; Alvarez-Sieiro et al., 2016; Ahmad et al., 2017). Bacteriocins are classified according to their common characteristics (Alvarez-Sieiro et al., 2016). Different post-translational modification enzymes and accessory proteins encoded along with the structural antimicrobial protein in the gene cluster of the bacterial genome are required for the biosynthesis of different classes of biologically active bacteriocins (Perez et al., 2014). The conservation of post-translational modification enzymes and accessory proteins in the biosynthetic gene clusters, as well as conserved motifs in the leader peptide of bacteriocin, allow for the discovery of novel bacteriocin encoded in the bacterial genome using genome mining (Scheffler et al., 2013; Boddy, 2014). Although less common, certain proteins and peptides without bacteriocin characteristic are also produced by bacteria, such as histone-like DNA binding and ribosomal proteins (Carvalho et al., 2010). Currently, only two bacteriocins have been approved by the Food and Drug Administration for human use, namely nisin and pediocin, which are produced by *Lactococcus lactis* subsp. *lactis* and *Pediococcus acidilactici*, respectively. Therefore, extensive research is required to discover new antimicrobial proteins. The screening and identification of new antilisterial proteins can be conducted using methods such as genome mining and mass spectrometry.

Genome mining involves the *in silico* identification of biosynthetic gene cluster of antimicrobial peptides in the whole or draft genome sequence of the microbes using bioinformatics tools, followed by *in vitro* analysis of the gene products (Scheffler et al., 2013). Recombinant protein expression followed by *in vitro* antimicrobial analysis can confirm the activity of target putative proteins, complementing any *in silico* approach (Scheffler et al., 2013).

The current study aims to identify potential antimicrobial proteins from a local milk curd isolate through genome mining and mass spectrometry. Several potential antimicrobial proteins contributing to the antimicrobial activity were discovered. The partially purified active fractions were tested against *L. monocytogenes*, and fractions displaying antilisterial activity were then subjected to mass spectrometry-based protein identification. The verification of antilisterial activity of the putative proteins was conducted via heterologous protein expression in *Escherichia coli* (*E. coli*) and characterizations of the recombinant proteins *in vitro*.

## Materials and Methods

### Bacterial Strain and Culture Condition

An unidentified pure bacterial culture exhibiting antilisterial activity previously isolated from milk curd was obtained from the Bioprocessing and Biomanufacturing Research Centre, Faculty of Biotechnology and Biomolecular Sciences, Universiti Putra Malaysia. It was grown in M17 broth at 37°C overnight. Repeated streaking and sub-culturing were carried out 10 times on M17 agar to obtain a single colony.

Additionally, *E. coli* T7 Express LysY/Iq cells (New England BioLabs Inc., Ipswich, Massachusetts, USA) were grown on Luria Bertani (LB) medium by overnight shaking (200–300 rpm) at 37°C in an incubator shaker (Heidolph Inkubator 1000). *L. monocytogenes* ATCC 15313 was cultured in Brain Heart Infusion (BHI) broth at 37°C overnight.

### Morphological and Biochemical Characterizations of Isolate

The isolate was subjected to Gram staining and biochemical tests. Catalase, oxidase, indole, nitrate reduction, and Voges Proskauer tests were carried out according to accredited methods at Bacteriology Laboratory of Veterinary Laboratory Service Unit, Faculty of Veterinary, Universiti Putra Malaysia.

### Identification of Bacterial Isolate Using Sanger Sequencing of Partial 16S rDNA Sequence

Species identification of the isolate was initially carried out using partial 16S rDNA sequence analysis. Partial 16S rDNA sequence was amplified using 27F and 1490R primers in 1X TopTaq Master Mix (Qiagen, Hilden, Germany). PCR was conducted using the following condition 95°C 3 min, 35 cycles of 95°C 1 min, 51°C 30 s, and 72°C 1 min, and a final extension at 72°C for 10 min was used on Mastercycler Gradient (Eppendorf, Hamburg, Germany). The amplicon was electrophoresed on 1% agarose gel with 1X Tris/Borate/EDTA (TBE) as a running buffer at 100 V for 20 min. ExactMark 1 kb DNA ladder 250–10,000 bp (Axil Scientific Pte Ltd, Singapore) was used as a marker. Upon visualization using FluorChem Gel Documentation System (Alpha Innotech, San Leandro, California, USA), the amplicon was sent for bidirectional Sanger sequencing (Axil Scientific Pte Ltd) using 27F and 1490R primers. The partial 16S rRNA gene sequence was analyzed against available non-redundant sequences in National Center for Biotechnology Information (NCBI) database using the Basic Local Alignment Search Tool Nucleotide (BLASTn). Default configuration was used for analysis.

### *In vitro* Antimicrobial Activity Assay

An *in vitro* antimicrobial activity assay was conducted using agar well diffusion method (Abbasiliasi et al., 2012) against *L. monocytogenes* ATCC 15313 as the indicator strain. The assay was performed in triplicate. Inhibition zones (in mm) were measured, and the value was used to calculate antimicrobial activity (AU) of the sample. Antimicrobial activity (AU) is expressed as the unit area of inhibition zone per unit volume of sample loaded into the well (mm^2^/mL) (Abbasiliasi et al., 2012).

### Whole-Genome Sequencing of *Paenibacillus polymyxa* Kp10

Genomic DNA was extracted from overnight-grown *Paenibacillus polymyxa* Kp10 (*P. polymyxa* Kp10) culture using the Wizard® Genomic DNA Purification Kit (Promega, Madison, USA) as per the manufacturer's protocol. An indexed paired-end library was constructed using 1 ng DNA of *P. polymyxa* Kp10 and Nextera XT DNA Library Prep Kit (Illumina Inc., San Diego, California, USA) according to the manufacturer's protocol. There were 151 base-pair paired-end (2 × 151 bp) sequencing reads obtained using MiSeq™ system (Illumina Inc., San Diego, California, USA) and were scanned for adapter sequences and low-quality sequences using BBDuk (BBTools version 36) (Bushnell and Brian, 2014). *De novo* genome assembly was carried out using SPAdes version 3.9.0 (Bankevich et al., 2012). NCBI Prokaryotic Genome Annotation Pipeline (Tatusova et al., 2016) was used to annotate the genome.

### Species Identification

The similarity of complete 16S rRNA gene sequence of the isolate obtained from the whole genome sequencing was analyzed against the available non-redundant sequences in the National Center for Biotechnology Information (NCBI) database using Basic Local Alignment Search Tool Nucleotide (BLASTn). Default configuration was used for the analysis. Additionally, the draft genome sequence of the isolate was analyzed using TrueBac™ID genome-based bacterial identification system (ChunLab Inc., Seoul, Korea) (Ha et al., 2019) to estimate the overall genome relatedness index (OGRI) against genome sequence of bacterial species that share more than 98.7% identity with the 16S rRNA gene sequence of the isolate.

### Mining the Draft Genome Sequence of *P. polymyxa* Kp10 for Antimicrobial Genes Using Bioinformatic Analysis

The draft genome sequence of *P. polymyxa* Kp10 was analyzed using BAGEL4 (Van Heel et al., 2018) and antiSMASH 5.0 (Medema et al., 2011) using default parameters. A Conserved Domain (CD)-Search (Marchler-Bauer et al., 2017) of putative biosynthetic proteins of the predicted antimicrobial proteins was carried out to verify their function based on the conserved domain. Amino acid sequences of <10 kDa proteins were also subjected to CD-Search using the default configuration to identify proteins with histone-like DNA binding protein domains. Accession numbers of contigs and locus tags of putative biosynthetic proteins were obtained from the Kp10's draft genome sequence data deposited in NCBI Genbank (PRJNA449134), which was updated as of April 2020.

### Partial Purification of Antilisterial Proteins From the Cell-Free Culture Supernatant of *P. polymyxa* Kp10

A 1 L overnight culture of *P. polymyxa* Kp10 was centrifuged (10,000 × g, 24°C for 5 min) to obtain cell-free culture supernatant (CFCS). Total proteins from CFCS were precipitated with the gradual addition of 790 g ammonium sulfate at 24°C. Following overnight incubation at 4°C, the precipitated protein was sedimented through centrifugation (12,000 × g, 4°C for 30 min) and dissolved in 50 mL sterile deionized distilled water. High molecular weight proteins were removed using Amicon® Ultra-15 Centrifugal Filter Unit with Nominal Molecular Weight Limit (NMWL) of 10 kDa (Merck, USA) through centrifugation (4,000 × g at 25°C for 10 min). The eluent (lower fraction) was then concentrated using Amicon® Ultra-15 Centrifugal Filter Unit with NMWL of 3,000 Da (Merck, USA) through centrifugation (4,000 × g at 25°C for 45 min). The resulting upper fraction was desalted using Hi Trap Desalting column before being further purified using Hi Trap SP HP cation exchange column on ÄKTA Purifier System (GE Healthcare, Chicago, Illinois, USA). The column was equilibrated with Tris-Cl buffer (pH 7.5) at a flow rate of 1 mL/min, and the proteins were eluted in 1 mL fractions using a sodium chloride gradient (0% to 100% of 1 M) in Tris-Cl buffer (pH 7.5). The protein concentrations of each fraction were monitored at 215 nm throughout purification.

Protein concentrations of all unbound and eluted fractions were determined using NanoOrange Protein Quantitation Kit (Invitrogen, Paisley, UK) according to the manufacturer's protocol. *In vitro* antilisterial activity of all unbound and eluted fractions was determined using the agar well diffusion method (Abbasiliasi et al., 2012). Partial purification, protein concentration determination, and *in vitro* antilisterial activity was carried out in triplicates.

### Sodium Dodecyl Sulfate-Polyacrylamide Gel Electrophoresis

Sodium dodecyl sulfate-polyacrylamide gel electrophoresis (SDS-PAGE) of the resulting protein fractions was performed using a precast 16.5% Mini-PROTEAN® Tris-Tricine Gel (BioRad, Hercules, USA). A 15 μL aliquot of Precision Plus Protein™ Dual Xtra Prestained Protein Standards (Bio-Rad, Hercules, USA) was used as molecular weight marker. An amount of 15 μg of protein samples was dissolved in 100 μL tricine sample buffer (200 mM Tris-HCl, pH 6.8, 40% glycerol, 2% SDS, 0.04% Coomassie Blue G-250) containing 2% fresh β-mercaptoethanol. The sample mixture was heated at 95°C for 5 min. Electrophoresis was conducted using tricine running buffer (100 mM Tris, 100 mM Tricine, 0.1% SDS, pH 8.3) at 100 V for 100 min. The SDS-PAGE gel was fixed using a fixative solution (40% methanol, 10% acetic acid) for 30 min, followed by staining using 0.025% (w/v) Coomassie Blue G-250 in 10% acetic acid for 1 h. The gel was then destained for 15 min in 10% acetic acid. The gel was viewed using a GS-800™ Calibrated Imaging Densitometer (Bio-Rad, Hercules, USA).

### Identification of Antilisterial Proteins Using nanoLC-MS/MS

A 1 mg/mL eluted protein displaying antilisterial activity in 0.1 M ammonium bicarbonate was digested by adding 100 μL of 0.05% Rapigest™ SF (Waters Corporation, Massachusetts, USA). After heat treatment at 80°C for 15 min, dithiothreitol with a final concentration of 100 mM was added, followed by incubation at 37°C for 30 min. Iodoacetamide with a final concentration of 200 mM was then added and incubated at room temperature for 45 min. An amount of 1 μg of trypsin powder was then added and further incubated at 37°C for overnight. A volume of 1 μL concentrated trifluoroacetic acid was added to stop digestion. The reaction mixture was centrifuged at room temperature (20,800 × g for 10 min). Then, 100 μL of supernatant was analyzed using LC Dionex 3000 Ultimate™ RSLCnano (Thermo Fisher Scientific) with A (0.1% formic acid in water) and B (0.1% formic acid in acetonitrile) as mobile phase. Peptides were separated on gradient basis: 5 to 40% B for 91 min, 2 min to 95% of B, 6 min at 95% of B, back to 5% of B in 2 min at the flow rate of 250 nL/min. EASY-Spray Column Acclaim™ PepMap™ C18 (100 Å, 2 μm particle size, 50 μm id × 15 cm) was used as separation column at 40°C. Next, 2 μL of protein sample was injected into Mass Spectrometer Orbitrap Fusion (Thermo Fisher Scientific) operated in data-dependent mode. Full scan spectra were collected (OTMS1) using the following parameters: scan range 310–1,800 m/z, resolving power of 120,000, automatic gain control (AGC) target of 4.0e^5^ (400,000), and maximum injection time of 50 ms. The method consisted of 3 s Top Speed Mode where precursors were selected for a maximum 3 s cycle. Only precursors with an assigned monoisotopic m/z and a charge state of 2–7 were further analyzed for MS2. All precursors were filtered using a 20 s dynamic exclusion window and an intensity threshold of 5000. The Ion Trap MS2 spectra (ITMS2) were analyzed using the following parameters: rapid scan rate with a resolving power of 60,000; AGC target of 1.0e^2^ (100); 1.6 m/z isolation window; and a maximum injection time of 250 ms. The precursors were fragmented by collision-induced dissociation (CID) and high energy collision dissociation (HCD) at normalized collision energy of 30 and 28%. Thermo Scientific™ Proteome Discoverer™ Software (Version 2.1) was used to analyze the data. The resulting output was used to search against the draft genome sequence of *P. polymyxa* Kp10 (Bioproject Accession: PRJNA449134;) with the following parameters: Missed cleavage: 2, MS1 tolerance: 10 ppm, MS2 tolerance: 0.6 Da including variable modification: Oxidation (M), deamidation of asparagine (N), and glutamine (Q) and Fixed modification: Carbamidomethyl (C). All peptides were validated using the percolator® algorithm, based on q-value <1% False Discovery Rate (FDR). The nanoLC-MS/MS analysis of the active fraction was repeated three times.

### Cloning and Heterologous Expression of Putative Antilisterial Proteins in *Escherichia coli* T7 Express LysY/Iq

Genes encode for putative antilisterial proteins P1, P2, P3, and P4 were amplified from the genomic DNA of *P. polymyxa* Kp10 using the primers listed in Table S1 to incorporate enterokinase recognition site at the 3' end. *Nco*I and *Xho*I restriction sites were used to flank the amplified DNA fragment at 5' and 3' end, respectively, to allow directional cloning into pET28b+ (Novagen). Upon transformation into *E. coli* T7 Express LysY/Iq (New England BioLabs, Inc) according to manufacturer's protocol, the positive transformants were selected on LB agar containing 30 μg/mL of kanamycin. Positive transformants of the respective constructs were confirmed using colony PCR and sequencing. Upon verification, the positive clones were designated as pET28b_P1, pET28b_P2, pET28b_P3, and pET28b_P4.

The expression of recombinant antilisterial proteins was initiated by inoculating 10 mL LB broth containing 30 μg/mL kanamycin with single colonies of positive clones and grown at 37°C until OD_600_ reached 0.4. The *E. coli* cells containing each recombinant plasmid (pET28b_P1, pET28b_P2, pET28b_P3, and pET28b_P4) were cultured in triplicates. Expression was then induced by 0.4 mM isopropyl β-D-1-thiogalactopyranoside (IPTG) followed by incubation at 37°C for 2 h. Proteins from the cell pellets were extracted using xTractor Buffer (Clontech Laboratories, Inc., Mountain View, USA) according to the manufacturer's protocol. The amount of total protein was estimated using the NanoOrange Protein Quantitation Kit (Invitrogen, Paisley, UK). *In vitro* antimicrobial activity of the recombinant antilisterial proteins was then determined using agar well diffusion assay (Abbasiliasi et al., 2012). For respective proteins, each well was loaded with the crude recombinant proteins obtained from three different overnight cultures as biological replicates. CFCS of Kp10 was used as positive control in the assay.

## Results and Discussion

### Biochemical and Antimicrobial Activity Characterizations of Isolate and Bacterial Identification Using Partial 16S rDNA Sequence Analysis

Besides *Bacillus* (Abriouel et al., 2011; Barbosa et al., 2015) and *Lactobacillus* (Messaoudi et al., 2013; da Silva Sabo et al., 2014), *Paenibacillus* (Aleti et al., 2016; Cochrane and Vederas, 2016) is another genus of bacteria with a rich resource of genes encodes for novel antimicrobial proteins and secondary metabolites. The CFCS of an unidentified pure bacterial culture isolated from milk curd has preliminarily been found to confer high antilisterial activity at 1051.84 ± 11.84 AU (Figure S1).

Biochemical characteristics of *P. polymyxa* Kp10 in comparison to that of other *Paenibacillus* species identified by other researchers are summarized in Table S2. Gram staining of the pure isolate shows purple-stained bacteria with a rod-shape morphology. The isolate was positive in catalase and acetyl-methyl carbinol production and was negative in oxidase and indole test. The positive reduction of nitrate showed that the isolate produced nitrate reductase. The biochemical characteristics of this isolate are similar to *P. polymyxa* OSY-DF (He et al., 2007) with the exception of acetyl-methyl carbinol production property that was not determined in the authors' study.

BLASTn analysis of the partial 16S rDNA sequence of the bacterium amplified using 27F and 1490R primers (Song et al., 2016) found that it shared 99% identity to *P. polymyxa* CF05 (Accession: CP009909.1), *P. polymyxa* SQR-21 (Accession: CP006872.1), *P. polymyxa* HY96-2 (Accession: CP025957.1), *P. polymyxa* Sb3-1 (Accession: CP010268.1), and 98% identity to *Paenibacillus sp* Izh-N1 (Accession: CP025696.1), with expectation value of 0.1 and query coverage of 98%. However, partial 16S rDNA sequence is insufficient to define the taxonomy of bacteria (Chun et al., 2018). A combination of the complete 16S rDNA sequence similarity and OGRI has been suggested to infer prokaryotic taxonomy (Chun et al., 2018). Hence, a high-quality draft of the bacterial genome sequence of *P. polymyxa* Kp10 was also obtained.

### Whole-Genome Sequencing and Molecular Identification of *P. polymyxa* Kp10

General genome features of the isolate are summarized in Table S3. The sequencing using MiSeq (Illumina) generated 6,154,868 paired reads with length ranging from 35 to 151 nucleotides. A total of 4,554,785 paired reads were obtained after removal of low-quality reads and adapter sequences. *De novo* genome assembly generated 122 contigs with a total genome size of 5,653,475 bp. The longest contig length is 1,294,617 bp and the average G+C content is 45.47%. N50 value of the genome was 619,841 bp with estimated assembly coverage of 158.28X. The genome annotation of the draft genome sequence using the NCBI Prokaryotic Genome Annotation Pipeline revealed a total of 5,054 genes including 4,808 protein-coding genes, and 124 RNA genes. The Whole Genome Shotgun project has been deposited to NCBI with GenBank assembly accession no: GCA_003052505.1 and BioProject accession no: PRJNA449134. Based on the minimal standards proposed by Chun et al. (2018) for prokaryotes taxonomy, BLASTn analysis of the full length 16S ribosomal RNA sequence of the antilisterial isolate shows 100% identity with *P. polymyxa* CF05 (Accession: CP009909.1) and *P. polymyxa* HY96-2 (Accession: CP025957.1) with expectation value of 0.0 and query coverage of 100%. TrueBac™ID genome analysis successfully identified Kp10 as a genuine *P. polymyxa* by 98.33 and 91.3% of ANI and ANI coverage, respectively, to *P. polymyxa* ATCC842. This online identification tool revealed 99.8, 99.62, and 99.52% of 16S rRNA, *recA*, and *rplC* sequence similarity, respectively, to *P. polymyxa*. In another study, the draft genome sequence of Kp10 was included in a comparative genomic analysis with other 49 selected *Paenibacillus* strains. A dendrogram based on ANI matrix has disclosed that Kp10 belongs to the same cluster with *P. polymyxa* ATCC 842, which was classified as an original *P. polymyxa* species (Jeong et al., 2019). In this work, the strain designation of Kp10 was given to this bacterium to ease future reference.

### Mining the Draft Genome Sequence of *P. polymyxa* Kp10 for Antimicrobial Genes and Biosynthetic Gene Clusters Using Bioinformatic Analysis

**Figure 1:**
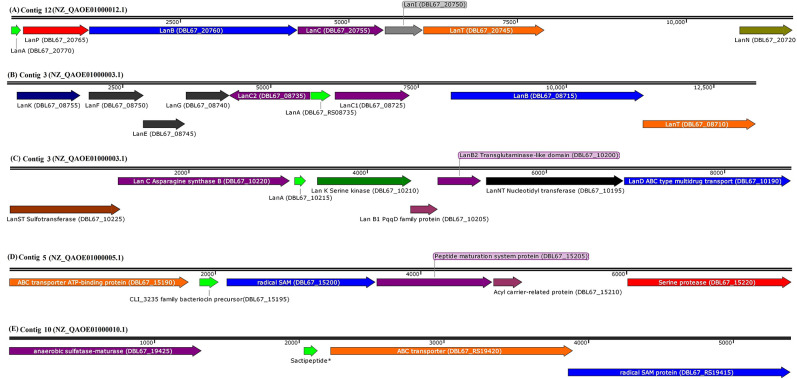
Arrangement of genes encode for structural peptide and putative biosynthetic proteins in biosynthetic gene cluster of **(A)** paenibacillin (GenBank coordinates from 63008 to 75697 in complementary strand), **(B)** paenilan (GenBank coordinates from 26187 to 38676 in complementary strand), **(C)** paeninodin (GenBank coordinates from 328300 to 337092 in complementary strand), **(D)** CLI_3235 family bacteriocin precursor, predicted as sactipeptide (*sacti5*) by BAGEL4 (Genbank coordinates from 183766 to 191369 in forward strand), and **(E)** sactipeptide* (GenBank coordinates from 68257 to 73648 in complementary strand) in respective contigs of *P. polymyxa* Kp10 genome. Sactipeptide* of the contig 10 (*sacti10*) has failed to be annotated by NCBI Prokaryotic Genome Annotation Pipeline. BAGEL4 analysis annotates the open reading frame as sactipeptide. The contigs with their respective accessions and locus tags of each genes/coding sequences are included in the figure. Genes are shown to scale and coordinates are relative to the length of each segment.

Bioinformatic analysis using BAGEL4, antiSMASH, and CD-Search successfully predicted eight antimicrobial peptides in the genome sequence of Kp10. The amino acid sequences of the antimicrobial peptides are presented in Table 1, and their biosynthetic gene clusters and putative functions are described in Table S4. Arrangement of genes encode for structural peptides and putative biosynthetic proteins in biosynthetic gene cluster of Kp10 is illustrated in Figure 1. BAGEL4 and antiSMASH analysis detected the existence of biosynthetic gene clusters responsible for the production of six ribosomally-synthesized antimicrobial proteins, namely two lantibiotics (predicted as paenibacillin and paenilan), one lasso peptide (predicted as paeninodin), one predicted thiazole/oxazole modified microcins (TOMM) peptide, and two sactipeptides (designated as *sacti5* and *sacti10*). The functions of the putative biosynthetic proteins encoded at the upstream and downstream of the precursor gene were further verified through conserved domain analysis using CD-Search (Table S4). In comparison to the extensively studied paenibacillin, paenilan, and paeninodin produced by *Paenibacillus polymyxa* OSY-DF (He et al., 2008), *Paenibacillus polymyxa* E681 (Park et al., 2017), and *Paenibacillus dendritiformis* C454 (Zhu et al., 2016), respectively, characterization and determination of biosynthesis mechanisms of TOMM and sactipeptide in other *Paenibacillus polymyxa* received less attention by the researchers. TOMM is featured by the presence of thiazole and oxazole heterocycles derived from modification of cysteine and serine residues (Metelev and Ghilarov, 2014).

**Table 1:** Potential antimicrobial peptides or proteins identified in the draft genome sequence of *Paenibacillus polymyxa* Kp10 using BAGEL4^a^, antiSMASH^b^, and CD-Search^c^.

**No**	**Predicted antimicrobial peptides (Protein ID/Locus tag)**	**Location (contig number)**	**Amino acid sequence**	**Theoretical isoelectric point (pI)**	**Theoretical molecular weight (Da)**
1	Lanthipeptide paenibacillin^a,b^ (WP_080561132.1/ DBL67_20770)	12	MKVDQMFDLDLRKSYEASELSPQ**ASIIKTTIKVSKAVCKTLTCICTGSCSNCK**	9.27	3105.79
2	Lanthipeptide paenilan^a,b^ (WP_019686785.1/DBL67_RS08735)	3	MKNQFDLDLQVAKNEVAPKEVQPA**SGLICTPSCATGTLNCQVSLSFCKTC**	7.57	2638.10
3	Lasso peptide paeninodin^b^ (WP_013369856.1/ DBL67_10215)	3	MSKKEWQEPTIEVLDINQTM**AGKGWKQIDWVSDHDADLYNPS**	4.56	2502.68
4	*Sacti5*/Sactipeptide^a^ (WP_080561119.1/DBL67_15195)	5	MRKLVKRSTNVGDTIEAFG**CGCSCYCPCSCYCAGSLTRSSNTSRESDGSYRRDNGTGIGNY**	7.73	4455.81
5	*Sacti10*/Sactipeptide^a^ (Not being annotated)	10	MDVLVKSAVQVSA**HCPFHGSSGCTLSCSYKQ**	7.97	1942.17
6	Thiazole/oxazole-modified microcins ^b^ (PTU47115.1/DBL67_10885)	3	MATEVLQTQVIQKAWEDASFREKLMADPKSAIRDVLGVVIPDHIQIKTVEETSDQFYLVIPPNPSGVLATSQKPRSMW	5.18	8781.12
7	Histone-like DNA binding protein HU^c^ (PTU44600.1/DBL67_22290)	15	MNKTDLINNISSKSGLSKRDVEAVLNGVLGEITDALASGDKVQLIGFGTFETRKRSSRTGRNPQTGNTIEIPESTVPAFKAGNKLKEAVN	9.52	9603.83
8	Histone-like DNA binding protein HU^c^ (PTU48889.1/DBL67_03415)	1	MLNKTDLINQVSESTELSKKDVTKAIDAVFEAIAGALQNGDKVQLVGFGNFEVRERSARKGRNPQTGEEIEIPASKIPAFKPGKALKDGIK	9.00	9854.26

*Theoretical isoelectric point and molecular weight of mature peptides (1–5; bold) were computed using Compute pI/MW tool of ExPAsy (Bioinformatics Resource Portal). Cleavage site of paenibacillin, paenilan, and lasso peptide were determined based on previous research (He et al., 2008; Zhu et al., 2016; Park et al., 2017). Cleavage site of sactipeptides and thiazole/oxazole modified microcins were predicted using SignalP 5.0. There was no cleavage site predicted in thiazole/oxazole modified microcins and histone-like DNA binding protein HU*.

The gene encoding for biosynthetic proteins is absent both upstream and downstream of the TOMM precursor in Kp10. Nevertheless, the BLASTp analysis against non-redundant protein sequences database shows that it shares 100% identity to nitrile-hydratase leader peptide (NHLP)-related natural product precursor of *P. polymyxa* (WP_016821365.1). The sequence is conserved in multispecies of *Paenibacillus*. Quite recently, the emergence of NHLP-related peptide as a new precursor in TOMM family has been addressed (Haft et al., 2010; Melby et al., 2011; Cox et al., 2015). Post-translational modification of NHLP-related TOMM precursor is commonly carried out by dehydrogenase and cyclodehydratase, which introduces thiazole and oxazole heterocycles in the TOMM structure (Metelev and Ghilarov, 2014).

In the case of sactipeptide, BLASTp analysis shows that *sacti10* does not share any similarity with protein sequences available in the database (Table S4). This suggests the novelty of *sacti10* found encoded in Kp10. BLASTp of *sacti5* shows that it shares 100% identity to CLI_3235 family bacteriocin precursor (Figure 1), which is a Cys-rich putative bacteriocin precursor peptides that commonly found in *Clostridia*. Radical SAM protein is commonly found encoded downstream of the CLI_3235 family bacteriocin precursor (Haft and Basu, 2011). The thioether bond between sulfur and α-carbon is a unique and distinctive characteristic of sactipeptide (Grove et al., 2017). Zhao et al. (2018) identified several putative biosynthetic gene clusters encode for sactipeptides in *Paenibacillus larvae, Paenibacillus odorifer, Paenibacillus graminis, Paenibacillus riograndensis*, and *Paenibacillus sp*. through bioinformatic analysis. However, the detailed amino acid sequence of the protein was not disclosed.

In addition to the two lantibiotics, one lasso peptide, one TOMM peptide, and two sactipeptides predicted by BAGEL4 and antiSMASH, CD-Search of proteins <10 kDa identified two different proteins with histone-like DNA binding protein HU domain (Table 1 and Table S4). The histone-like DNA binding proteins were not predicted by BAGEL4 and antiSMASH.

### Partial Purification of Antilisterial Proteins From the CFCS of KP10 and Protein Identification Using Nano LC-MS/MS

**Figure 2:**
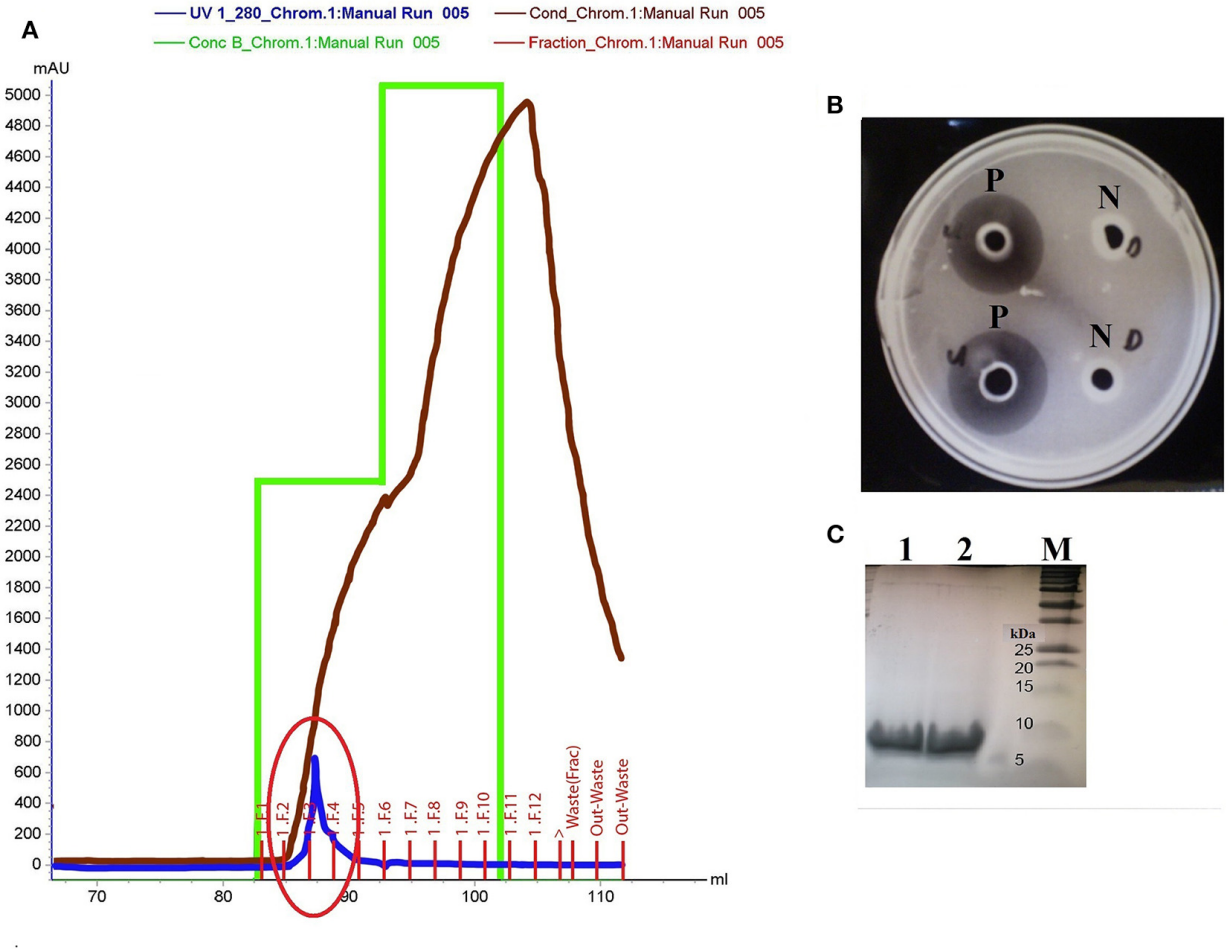
**(A)** Purification of active fraction using cation exchange chromatography (Hi Trap SP HP cation exchange column) on Akta Purifier System. The purification yields a single peak that covers F2, F3, and F4 fractions (red circle). **(B)** P: Antilisterial activity assay of the pooled active fractions determined using agar well diffusion method, analyzed in duplicates. N: There was no inhibition zone obtained in the control wells containing Tris-Cl buffer, pH 7.5. **(C)** SDS-PAGE of the active fraction mixture pooled from F2, F3, and F4 fractions analyzed in duplicates (Lane 1 and 2). Lane M: Precision Plus Protein™ Dual Xtra Standards (BioRad).

The *in silico* discovery of eight potential antimicrobial proteins from Kp10 indicates the antimicrobial potential of Kp10. Hence, we hypothesize the involvement of at least one of the proteins in the antilisterial activity of Kp10. In theory, all these proteins may not necessarily involve in the antilisterial property displayed by the CFCS of the isolate. The genes found in the genome may be silenced or constitutively/inductively expressed by the bacteria. Therefore, we carried out protein identification by analyzing the active protein fractions using nanoLC-MS/MS. In the initial attempt to purify the proteins, ammonium sulfate-precipitated proteins from the CFCS of Kp10 was subjected to separation based on molecular weight using Amicon® Ultra-15 Centrifugal Filter Unit with NMWL of 10 kDa and 3 kDa. Both the retentate and eluant were analyzed for *in vitro* antimicrobial activity. Antilisterial activity was only detected in the eluant of 10 kDa and retentate of 3 kDa, suggesting that the antilisterial protein has a size between 3 and 10 kDa. The final cation exchange chromatography step was carried out using Tris-Cl buffer at pH 7.5. Consecutive purification steps successfully yielded a single peak that covers F2, F3, and F4 fractions (Figure 2A). Antilisterial activity was successfully detected in the mixture of these three fractions with a total activity of 2.18 × 10^4^ ± 0 AU (Figure 2B). The purification recovery of the antilisterial proteins from each purification steps is summarized in Table S5. SDS-PAGE of the fractions' mixture yielded single electrophoretic bands between 5 and 10 kDa (Figure 2C).

The amino acid sequences of the predicted antimicrobial proteins obtained from the draft genome sequence of Kp10 (NZ_QAOE00000000.1) were used as a reference database in nanoLC-MS/MS analysis. Intriguingly, two proteins identified by the CD-Search of protein <10 kDa in the Kp10 genome; the histone-like DNA binding proteins were found in the active fractions. The two proteins were designated as P1 and P2 (Table 2). On the other hand, the other six predicted bacteriocins (paenibacillin, paenilan, paeninodin, TOMM, sactipeptides) previously identified *in silico* in the Kp10 genome were not detected in the active fractions. Besides the P1 and P2 proteins, two other proteins detected in the active fractions were translation initiation factor IF-1 (P3) and 50S ribosomal protein L29 (P4) (Table 2).

**Table 2:** Identification of proteins in active fraction mixture obtained from cation exchange chromatography using nanoLC-MS/MS.

**Protein designation**	**Protein name/Locus Tag**	**Sum PEP score**	**Coverage**	**PSMs**	**Unique peptides**	**AAs**	**MW [kDa]**	**Calc. pI**	**Score sequest HT**
P1	Histone-like DNA binding protein HU/DBL67_22290	2.78	21.11	2.00	1.00	90.00	9.60	9.52	5.41
P2	Histone-like DNA binding protein HU/DBL67_03415	2.66	15.38	3.00	1.00	91.00	9.85	9.00	9.40
P3	Translation initiation factor IF-1/DBL67_23515	12.64	76.06	26.00	4.00	71.00	8.02	8.53	71.03
P4	50S ribosomal protein L29/DBL67_23595	7.89	53.85	22.00	3.00	65.00	7.42	9.82	54.48

*PEP, Posterior Error Probability; Coverage, The percent coverage calculated by dividing the number of amino acids in all found peptides by the total number of amino acids in the entire protein sequence; PSMs, Peptide Spectrum Matches; AAs, number of amino acids found in the protein; MW [kDa], Theoretical Molecular weight in kDa; Calc. pI, calculated isoelectric point; Score Sequest HT, Sum of the scores of the individual peptides from the Sequest HT search. P1, P2, P3, and P4 are the designation given to each identified protein*.

### Confirmation of Putative Antilisterial Proteins by Cloning and Heterologous Expression in *E. coli*

Further verification on the antilisterial activity of the putative antimicrobial proteins was carried out by repeating the antilisterial activity assay using the recombinant proteins. The genes encoding for P1, P2, P3, and P4 were separately cloned into pET28b+ for expression in *E. coli* T7 Express LysY/I^q^ (New England BioLabs). PCR amplification of genes encode for P1, P2, P3, and P4 obtained amplicon of 305, 287, 227, and 209 bp in size, respectively (Figure S2A). The colony PCR of the transformants (pET28b_P1, pET28b_P2, pET28b_P3, and pET28b_P4) confirmed the successful cloning of the four genes into pET28b+ (Figure S2B).

**Figure 3:**
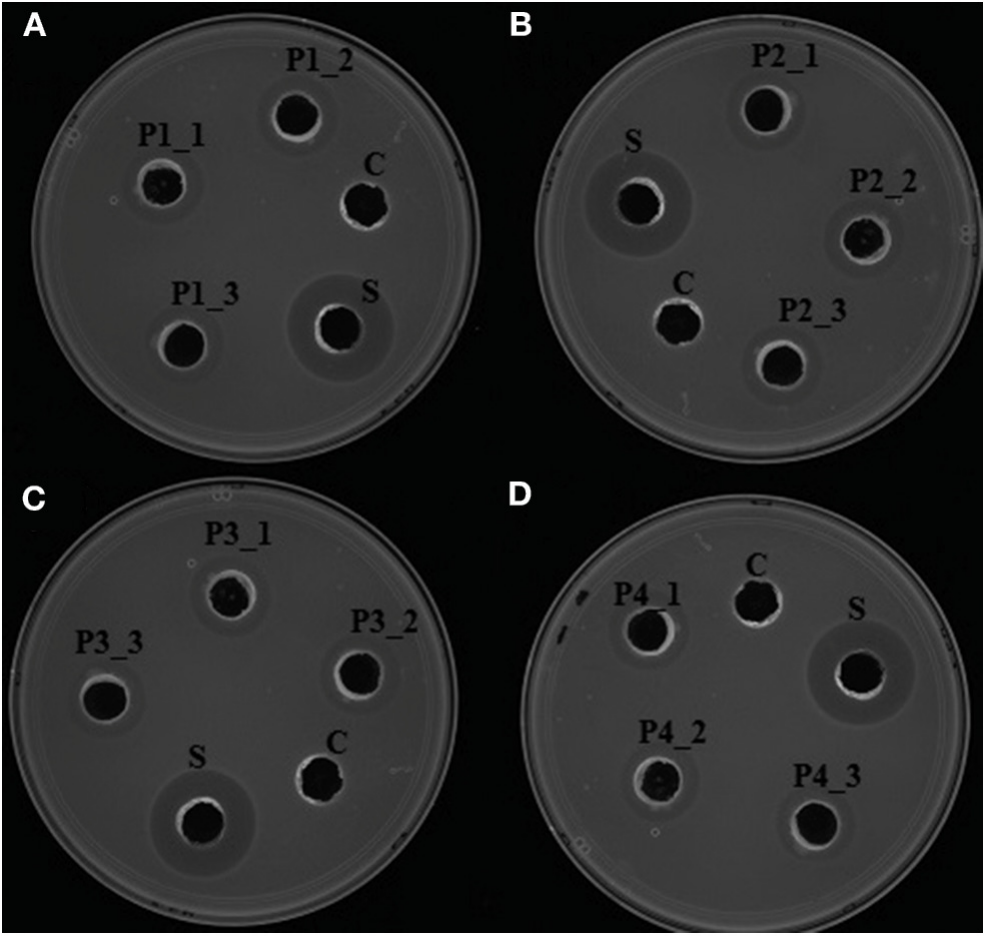
*In vitro* antimicrobial activity assay of recombinant antilisterial proteins. Each crude recombinant protein was obtained from three different cultures and assayed in triplicates. (i) **(A)** P1_1, P1_2, and P1_3, **(B)** P2_1, P2_2, P2_3, **(C)** P3_1, P3_2, P3_3, and **(D)** P4_1, P4_2, P4_3 using agar well diffusion assay. C: crude protein of untransformed *E. coli* T7 Express LysY/Iq (New England BioLabs, Inc). S: cell-free culture supernatant of *P. polymyxa* Kp10. Inhibition zones were obtained in all of the recombinant proteins, thus confirming their antilisterial activity.

Upon induction of protein expression and extraction, *in vitro* antimicrobial activity assay of the total crude protein containing the recombinant proteins conferred antilisterial activity (Figure 3). Recombinant P1, P2, P3, and P4 showed inhibition zones of 14±0 mm in diameter, corresponding to 1257.14 ± 0 AU/mL. There was no antilisterial activity detected in the crude protein of empty *E. coli* T7 Express LysY/Iq cultures. Pidutti et al. (2018) classified the strength of antibacterial activity of protein into three groups: strong (10–15 mm inhibition zone); moderate (5–10 mm inhibition zone); and no inhibitory activity (0–5 mm inhibition zone). Based on this classification, strong antilisterial activity was observed in all recombinant proteins.

The antimicrobial activity of the P1, P2, P3, and P4 proteins is not unprecedented. Carvalho et al. (2018) also identified several ribosomal proteins (30S ribosomal protein S19, S20, and S21; 50S ribosomal protein L24 and L29), one histone-like DNA binding protein HU, and one translation initiation factor IF-1 produced by *Lactobacillus sakei* subsp. *sakei* 2a with the ability to inhibit the growth of several pathogenic bacteria, including *L. monocytogenes* and *L. innocua*. In addition, Pidutti et al. (2018) discovered the antibacterial activity of 50S ribosomal protein L27 and L30 produced by *Lactobacillus salivarius* SGL 03 against *Enterococcus faecium* Sintal Group, *Streptococcus pyogenes* ATCC 19615, and *Staphylococcus uberis* ATCC700407. The antimicrobial mechanisms of these proteins have been proposed by several authors. Carvalho et al. (2018) suggested that the histone-like DNA binding protein disrupts the DNA structure of susceptible strain and halts DNA replication, in turn contributing to cell death. A study on the mechanism of action of antimicrobial histone-like DNA binding protein, translation initiation factor IF-1, and ribosomal proteins produced by *Lactobacillus sakei* subsp. *sakei 2a* found that these proteins caused dissipation of membrane potential but has no impact on pH gradient. Carvalho et al. (2010) suggest that these proteins do not cause cell leakage or cell lysis. The high isoelectric point of these proteins may trigger strong attraction towards a negatively charged phosphate group of nucleic acids at normal physiological pH. Similarly, Pidutti et al. (2018) showed that ribosomal proteins L27 and L30 of *Lactobacillus salivarius* SGL03 conferred bactericidal action against *Streptococcus pyogenes*. Due to the role of these proteins in DNA replication and the regulation of protein expression, they are constitutively expressed by all *P. polymyxa* strains. Hence, a dedicated secretion pathway is probably present specifically in Kp10 to allow the secretion of these proteins to the extracellular milieu, either through direct penetration or exocytosis (Le et al., 2017). Polycationic nature of these peptides could also trigger high affinity to the anionic surfaces of the bacterial cell wall. This interaction may enable the peptide to cross the lipid bilayer of the sensitive strains. However, this remains to be elucidated.

## Conclusion

This study determined four extracellular antilisterial proteins produced by Kp10, an isolate originating from milk curd. Eight different ribosomally-synthesized antimicrobial proteins were predicted *in silico*. Interestingly, two of the eight predicted proteins, namely histone-like DNA binding proteins (P1 and P2), were identified via mass spectrometry as being responsible for the antilisterial activity. In addition, one translation initiation factor IF-1 (P3) and one 50S ribosomal protein L29 (P4) were also found to contribute to the antilisterial activity of the Kp10. This was further confirmed by heterologous protein expression in *E. coli* and the antilisterial assay of the recombinant proteins against *L. monocytogenes*. Future studies on the mechanism of inhibition, factors that affect secretion, and the applicability of these proteins in food and medicine are called for. In the future, heterologous expression of the remaining antimicrobial proteins identified *in silico* in this study should be carried out, which increase the variability of antimicrobial protein reservoir to be explored further for food and medical industries.

## Data Availability Statement

Draft genome sequence of *P. polymyxa* Kp10 can be found in NCBI Repository with assembly accession no: GCA_003052505.1 and BioProject accession no: PRJNA449134.

## Author Contributions

The research was designed by NM, RAR, SM, and SA. The experiments were carried out by NM, AZ, SA, AAA, and RR. Data were analyzed and interpreted by NM, AM, and SA. SM, IH, AA, and RAR contributed reagents, materials and analysis tools. The manuscript was written by NM and revised by SA, AM, and IH. RAR, SM, and AM supervised and provided critical evaluation of the project. All authors had read and approved the final manuscript.

## Conflict of Interest

The authors declare that the research was conducted in the absence of any commercial or financial relationships that could be construed as a potential conflict of interest.
