# Supplementary material for: The Discovery of New Antilisterial Proteins From Paenibacillus polymyxa Kp10 via Genome Mining and Mass Spectrometry
Source: Front Microbiol. 2020 Jul 2;11:960. doi: 10.3389/fmicb.2020.00960 (PMC7343975; doi:10.3389/fmicb.2020.00960)
Supplement: Supplementary file 1 [file Data_Sheet_1.pdf]

1. Supplementary Data
2. Supplementary Figures and Tables
   2.1. Supplementary Figures

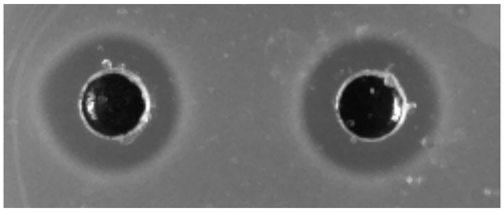

Figure S1. Inhibition zone obtained from agar well diffusion assay of the cell-free culture supernatant of an unidentified pure bacterial culture isolated from milk curd against *Listeria monocytogenes*

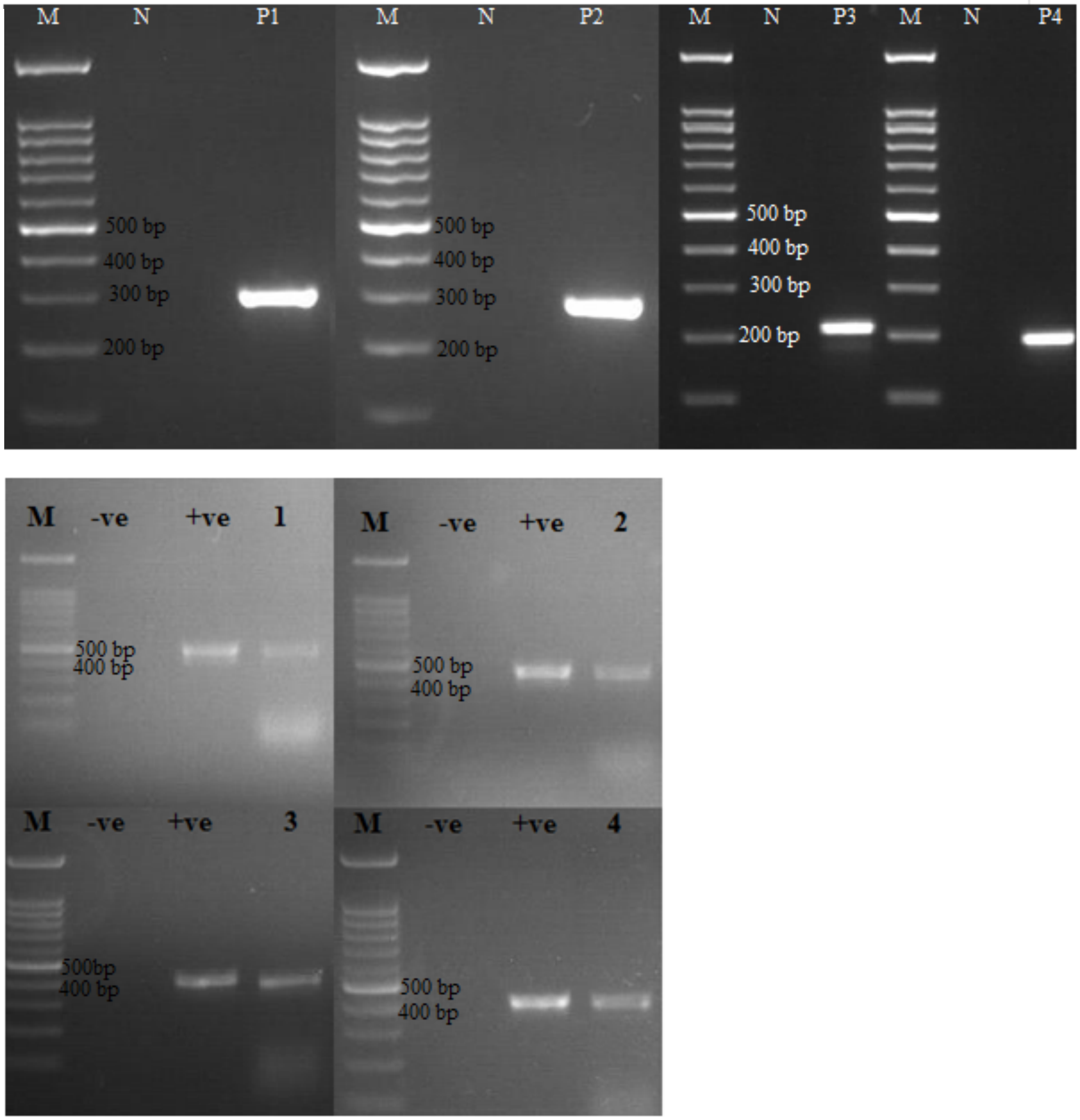

Figure S2. **(A)** PCR amplification of genes encode for P1, P2, P3 and P4. The amplicons were 305, 287, 213, and 195 bp in size, respectively. M: ExactMark 100 bp DNA ladder (Axil Scientific), P1, P2, P3, P4: Amplicon produced by PCR using P1, P2, P3 and P4 primers set, respectively, N: No template DNA in PCR. **(B)** Colony PCR of positive *E. coli* T7 Express LysY/Iq transformants (1) pET28b_P1, (2) pET28b_P2, (3) pET28b_P3 and (4) pET28b_P4. –ve: No PCR template; +ve: positive control (pET28b containing respective insert gene purified from *E. coli* NEB10); M: ExactMark 100 bp DNA Ladder, Ready-To-Use (BIO-5130)

2.2. Supplementary Tables

Table S1. Oligonucleotide sequence of primers used in this study. 27F and 1490R were used for amplification of partial 16S sequence; P1F-P1R were used for PCR amplification of putative antilisterial protein P1, P2F-P2R were used for PCR amplification of putative antilisterial protein P2, P3F-P3R were used for PCR amplification of putative antilisterial protein P3, P4F-P4R were used for PCR amplification of putative antilisterial protein P4, T7 Promoter Forward and T7 Terminator Reverse were used for colony PCR of positive transformants

Primer Set	Primer	Nucleotide sequence (5' to 3')
**16S**	27F	AGTTTGATCCTGGCTCAG
	1490R	GTTACCTTGTTACGACTTC
**P1**	P1F	CCATGGGCATGTTGAATAAAACGGATTTGATC
	P1R	GTCGACTTTAATTCCGTCTTTGAGCG
**P2**	P2F	CCATGGGCATGAACAAGACAGATCTGATC
	P2R	CTCGAGTTTGTCATCGTCATCGTCGACGTTAACGGCTTCTTTAAGC
**P3**	P3F	CCATGGGCATGAAGGCTAGTGAATTCCGCAAC
	P3R	GTCGACGCTAATCCCAAGTTCTCTTTCAC
**P4**	P4F	CCATGGGCTTGGCCAAAGAAGATGTCATTGAGG
	P4R	GTCGACCTTGCGGTACGTTATACGG
**Colony PCR**	T7 Promoter Forward	TAATACGACTCACTATAGG
	T7 Terminator Reverse	GCTAGTTATTGCTCAGCGG

Table S2. Biochemical characterizations of the *P. polymyxa* Kp10 for production of acetyl methyl carbinol, catalase, oxidase, indole and reduction of nitrate as compared to biochemical characteristics of other *Paenibacillus* species identified by other researchers. - Negative result; + Positive result; ND: Not determined

	Acetyl methyl carbinol	Catalase	Oxidase	Nitrate reduction	Indole	Reference
P. larvae	-	-	+	+	+	Hamdi et al., 2013
P. polymyxa ATCC842T	+	+	+	+	+	Hamdi et al., 2013
P. thiaminolyticus	-	+	+	-	-	Guo et al., 2012
P. polymyxa Kp10	+	+	-	+	-	This study
P. polymyxa OSY-DF	ND	+	-	+	-	He et al., 2007
Paenibacillus sp A3	-	-	-	ND	-	Baindara et al., 2016

Table S3. *Paenibacillus polymyxa* Kp10 genome features

Genome Feature	Value
Total length (bp)	5, 653, 475
No of contigs	122
Contig N50 (bp)	619, 841
Number of genes	5054
Number of coding sequences	4808
Number of RNA genes	124
Number of other ncRNA features	4
G+C content (%)	45.47

Table S4. Potential antimicrobial peptides or proteins and their biosynthetic proteins identified in *Paenibacillus polymyxa* Kp10 draft genome sequence through bioinformatic analysis using BAGEL4[a], antiSMASH[b] and CD-Search[c]. N/A: not applicable. N/D: not described

No	Predicted antimicrobial peptides (Protein ID/locus tag)	Predicted biosynthetic proteins/locus tag	CD-Search	Putative function	Location (contig number)
1	Lanthipeptide paenibacillin[a,b] (WP_080561132. 1/ DBL67_20770)	LanP/DBL67_20765	Peptidase S8 family domain (cd07492) of peptidase domain in S8 and S53 superfamilies (cl10459)	Contains conserved Asp/His/Ser catalytic triad for proteolytic cleavage of the leader peptide	Contig 12
		LanB/DBL67_20760	Lantibiotic dehydratase N-terminus family (pfam 04738)	Catalyzes dehydration of serine and threonine residues through glutamylation process	
			Thiopeptide-type bacteriocin biosynthesis domain (TIGR03891)	Crucial for the final glutamate elimination step	
		LanC/DBL67_20755	LanC domain-containing protein (cd04793) that encompasses:<br>(a) zinc-binding site (C C [HC])	Enables metal binding that is crucial for activation of the thiol substrate for nucleophilic addition	
			(b) active site (H C C [HC])	Catalyzes thioether formation via addition of cysteine thiols to dehydrolanine and dehydrobutyrine	
		LanT/DBL67_20745	MdlB family domain (COG1132)	ABC-type multidrug transport system for extracellular secretion of mature peptide	
		LanI/DBL67_20750	Not found	N/A	
		LanN/DBL67_20720	TraX superfamily (cl05434)	Amino terminal acetylation of mature peptide	

2	Lanthipeptide paenilan[a,b] (WP_019686785.1/ DBL67_RS08735)	LanB/DBL67_08715	Lantibiotic dehydratase N-terminus family (pfam 04738)	Catalyzes dehydration of serine and threonine residues through glutamylation process	Contig 3
			Thiopeptide-type bacteriocin biosynthesis domain (TIGR03891)	Crucial for the final glutamate elimination step	
		LanC1/DBL67_08725	LanC domain-containing protein (cd04793) that encompasses:<br>(a) zinc-binding site (C C [HC])	Enables metal binding that is crucial for activation of the thiol substrate for nucleophilic addition	
			(b) active site (H C C [HC])	Catalyzes thioether formation via addition of cysteine thiols to dehydrolanine and dehydrobutyrine	
		LanC2/DBL67_08735	LanC domain-containing protein (cd04793) that encompasses:<br>(a) zinc-binding site (C C [HC])	Enables metal binding that is crucial for activation of the thiol substrate for nucleophilic addition	
			(b) active site (H C C [HC])	Catalyzes thioether formation via addition of cysteine thiols to dehydrolanine and dehydrobutyrine	
		LanE/DBL67_08745	lantibiotic-exporting membrane permease, EfiE/EfiG/ABC2 family (COG4200).	ABC-type multidrug transport system and ATPase components that are crucial for defense mechanisms	
		LanF/DBL67_08750	ccm domain (COG1131)		
		LanG/DBL67_08740	lantibiotic-exporting membrane permease, EfiE/EfiG/ABC2 family (COG4200).		
		LanT/DBL67_08710	MdlB family domain (COG1132)	ATP binding cassette-type multidrug transport system for extracellular secretion of the mature peptide	

		LanK/DBL67_08755	Signal transduction histidine kinase (COG0642)	Functions as a protein kinase that phosphorylates a target protein in response to various signals.	
3	Lasso peptide paeninodin[b] (WP_013369856.1/ DBL67_10215)	LanB1/DBL67_10205	Coenzyme Pqq synthesis protein D family domain (pfam05402)	Binds to precursor peptide and directs it to LanB2	Contig 3
		LanB2/DBL67_10200	Transglutaminase-like domain (pfam13471)	Protease activity for removal of leader peptide	
		LanC/DBL67_10220	Glutamine amidotransferases class-II (GATase) asparagine synthase B (cd00712) of glucosamine 6-phosphate synthetase superfamily (cl28298)	For the formation of the Gly1-Glu8 amide bond that assists macrolactam ring formation	
		LanK/DBL67_10210	Phosphoenolpyruvate carboxykinase superfamily (cl22860)	Phosphorylates paeninodin precursor peptides at its C-terminal serine residue	
			Nucleoside/nucleotide kinase (NK) superfamily (cl17190)		
		LanD/DBL67_10190	MdlB domain	ABC-type multidrug transport system	
		LanNT/DBL67_10195	Uncharacterized nucleotidyltransferase (pfam14907) of nucleotidyltransferase (NT) domain of DNA polymerase beta and similar protein superfamily (cl11966)	N/D	
		LanST/DBL67_10225	Sulfotransferase family (pfam13469)	Catalyzes the transfer of sulfate from a donor to an acceptor	

4	*Sacti5*/Sactipeptide[a] (WP_080561119.1 /DBL67_15195)	Radical SAM /DBL67_15200	PRK13758 superfamily (cl28173) Cys-rich peptide radical SAM maturase CcpM<br><br>Members of this family are radical SAM enzymes that occur next to clostridial Cys-rich predicted bacteriocin	Radical SAM enzyme catalyzes reductive cleavage of SAM and producing 5-deoxyadenosyl radical. The 5-deoxyadenosyl radical catalyzes removal of hydrogen atom of the α-carbon of the acceptor amino acid. Intramolecular attack and transfer of electron to the previously deprotonated cysteine residue of the precursor peptide catalyze the formation of thioether bridge between the α-carbon and the sulfur group of cysteine residue	Contig 5
		Peptide maturation system protein/DBL67_15205	nat-prod-clost family (TIGR04066) of P-loop NTPase superfamily	As H^+-transporting two-sector ATPase and postulated to involve in bacteriocin maturation or immunity	
		Acyl-carrier related protein/DBL67_15210	ocin-ACP-rel family (TIGR04069)	Proposed putative function in biosynthesis of fatty acids and polyketides	
		Serine protease/DBL67_15220	Peptidase S8 family domain (cd07492) of peptidase domain in S8 and S53 superfamilies (cl10459)	Contains conserved Asp/His/Ser catalytic triad for proteolytic cleavage of the leader peptide	
		ABC transporter ATP binding protein/DBL67_15190	MdlB family domain (COG1132) ABC-type multidrug transport system, ATPase and permease component	Defense mechanism and extracellular secretion of the mature peptide	

5	*Sacti10*/Sactipeptide[a] (Not being annotated)	Anaerobic sulfatase maturase/DBL67_19425	Specific hit to PRK13758 superfamily (cl28173). Non-specific hit to TIGR04334 (PSSMID 213947) radical SAM/SPASM domain Clo7bot peptide maturase; In multiple strains of *Clostridium botulinum,* this single radical SAM domain protein occurs next to a tandem array of seven homologous Cys-rich small peptides (see TIGR04333). Because this radical SAM enzyme contains the SPASM domain, associated with peptide modification, it is proposed to modify all seven *C. botulinum* targets, hence named as Clo7bot for this system. Suggested gene symbol is ctpM (Clostridial Tandem Peptide Maturase).	Proposed function in protein modification and repair<br><br>Catalyzes thioether linkage between ß-carbon and the sulphur group of cysteine residue	Contig 10
		ABC transporter ATP-binding protein /DBL67_RS19420	ABC-type multidrug transport system, ATPase and permease component (COG1132)	Extracellular secretion of mature peptide<br><br>Defense mechanism	
		Radical SAM /DBL67_19415	Non-specific hit to Cys-rich peptide radical SAM maturase CcpM (TIGR04068) of PRK13758 superfamily (cl28173)<br><br>Members of this family are radical SAM enzymes that occur next to clostridial Cys-rich predicted bacteriocin	Radical SAM enzyme catalyzes reductive cleavage of SAM and producing 5-deoxyadenosyl radical. The 5-deoxyadenosyl radical catalyzes removal of hydrogen atom of the α-carbon of the acceptor amino acid. Intramolecular attack	

			and transfer of electron to the previously deprotonated cysteine residue of the precursor peptide catalyze the formation of thioether bridge between the α-carbon and the sulphur group of cysteine residue.	
6	Thiazole/oxazole-modified microcins [b] (PTU47115.1/ DBL67_10885)	Nitrile hydratase leader peptide (NHLP) domain family (TIGR03793) of TOMM_pelo super family (cl22942)	It is conserved among thiazole/oxazole-modified microcins (TOMMs) precursor<br><br>The domain is crucial for cleavage or extracellular secretion of the mature peptide	Contig 3
7	Histone-like DNA binding protein HU[c] (PTU44600.1 /DBL67_22290)	Histone-like DNA-binding protein HU (cd13831) of HU-IHF superfamily (cl00257)<br>Contains two conserved sites which are:<br>(1) dimer interface for polypeptide binding site<br>(2) DNA binding site for nucleic acid binding site	Binds non-specifically to duplex DNA with a preference to nicked and bent DNA	Contig 15
8	Histone-like DNA binding protein HU[c] (PTU48889.1 /DBL67_03415)			Contig 1

Table S5. Recovery of antilisterial proteins obtained in each purification steps. AU is defined as the unit area of inhibition zone per unit volume of sample loaded into the well (mm^2/mL)

	**Volume (mL)**	**Total protein (mg)**	**Total activity (AU)**	**Specific activity (AU/mg)**	**Fold purification (per step)**	**Yield (%) per step**
**Cell-free culture supernatant**	150	10.64±1.30	$2.98 \times 10^5 \pm 0$	$2.80 \times 10^4 \pm 3451$	1.00	100.00
**Ammonium sulphate precipitation**	50	14.29±1.15	$2.31 \times 10^5 \pm 0$	$1.62 \times 10^4 \pm 1305.2$	0.58	77.60
**Amicon Ultra Centrifugal Device 10 kDa and 3 kDa**	15	13.53±2.39	$6.94 \times 10^4 \pm 0$	$5.13 \times 10^3 \pm 1010.8$	0.18	30.00
**Cation exchange chromatography**	4	1.04±0.13	$2.18 \times 10^4 \pm 0$	$2.09 \times 10^4 \pm 2466.9$	4.07	31.38
